# Supplementary material for: SARS-CoV-2 infection induces adaptive NK cell responses by spike protein-mediated induction of HLA-E expression
Source: Emerg Microbes Infect. 2024 May 28;13(1):2361019. doi: 10.1080/22221751.2024.2361019 (PMC11212573; doi:10.1080/22221751.2024.2361019)
Supplement: Supplemental Material [file TEMI_A_2361019_SM3584.pdf]

Supplementary Figures

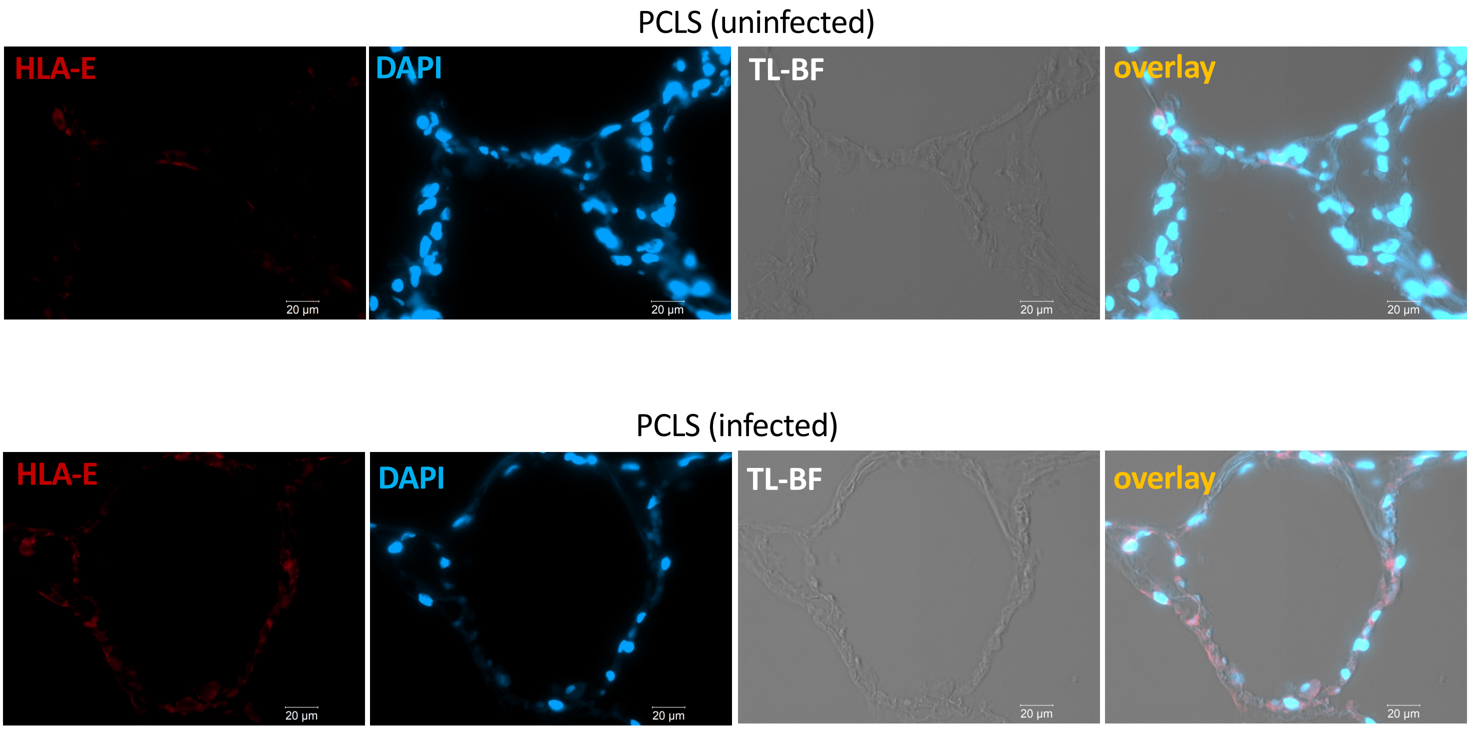

Fig. S1. Infection of human precision-cut lung slices (PCLS) with SARS-CoV-2 and analysis of HLA-E expression. Confocal laser scanning microscopy images of human precision-cut lung slices (PCLS) either uninfected (top) or infected with SARS-CoV-2, pango lineage B.1.513, (bottom). PCLS were stained with anti-HLA-E antibody (red) and stained for cell nuclei (DAPI, blue). Images were captured using plan-apochromat 63x/1.40 oil DIC M27 objective. Scale bar: 20 µm.

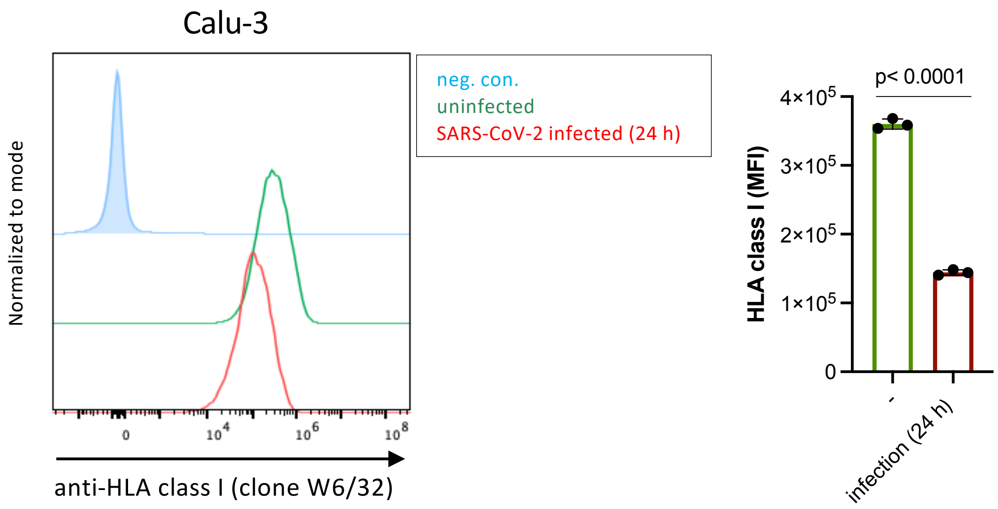

Fig. S2. Downregulation of classical HLA class I proteins upon infection with SARS-CoV-2.
Anti HLA class I monoclonal antibody (clone W6/32) was used for flow cytometry analysis of Calu-3 cells either untreated or infected with virus.

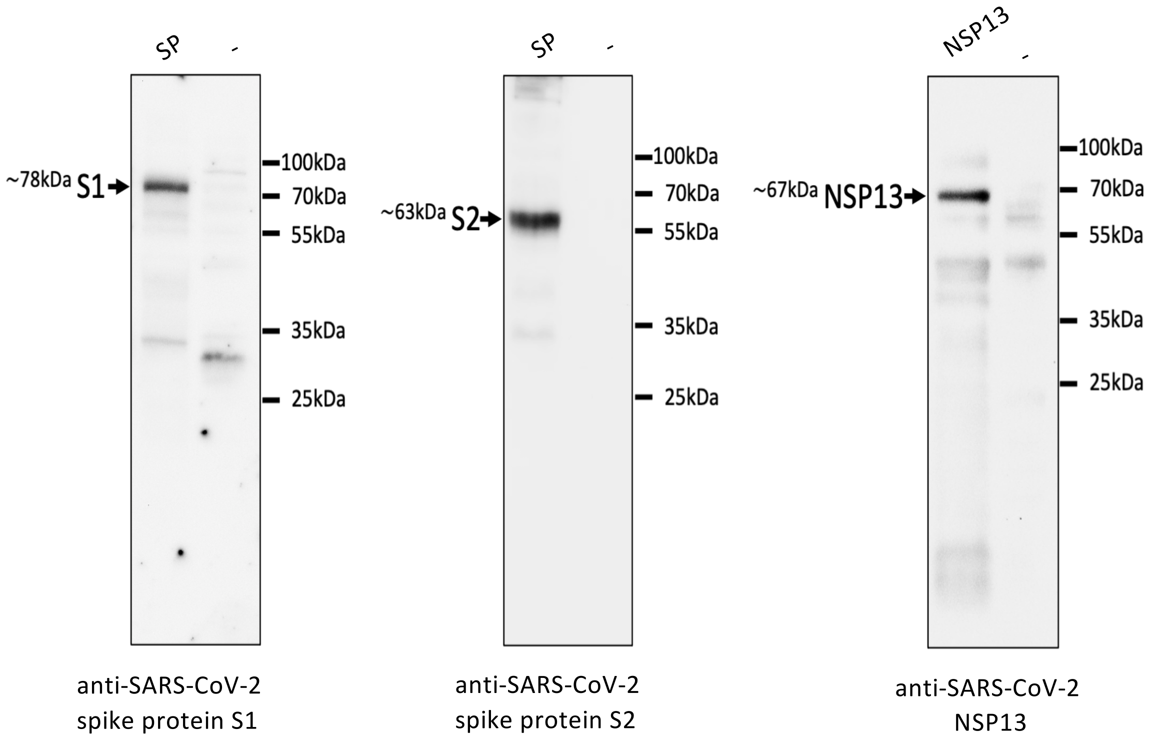

Fig S3. Western blot (WB) analysis of Calu-3 cells transfected with gene constructs expressing spike protein (SP) or NSP13. Subunits S1 and S2 of SP were detected in SP-transfected cell lysates using anti-SARS-CoV-2 SP S1 antibodies (left blot) or anti-SARS-CoV-2 SP S2 antibodies (middle blot). NSP13 was detected using an anti-SARS-CoV-2 NSP13 antibody (right blot). Cell lysates from untransfected cells are indicated (-). The expected molecular weights for S1, S2, and NSP13 are indicated.

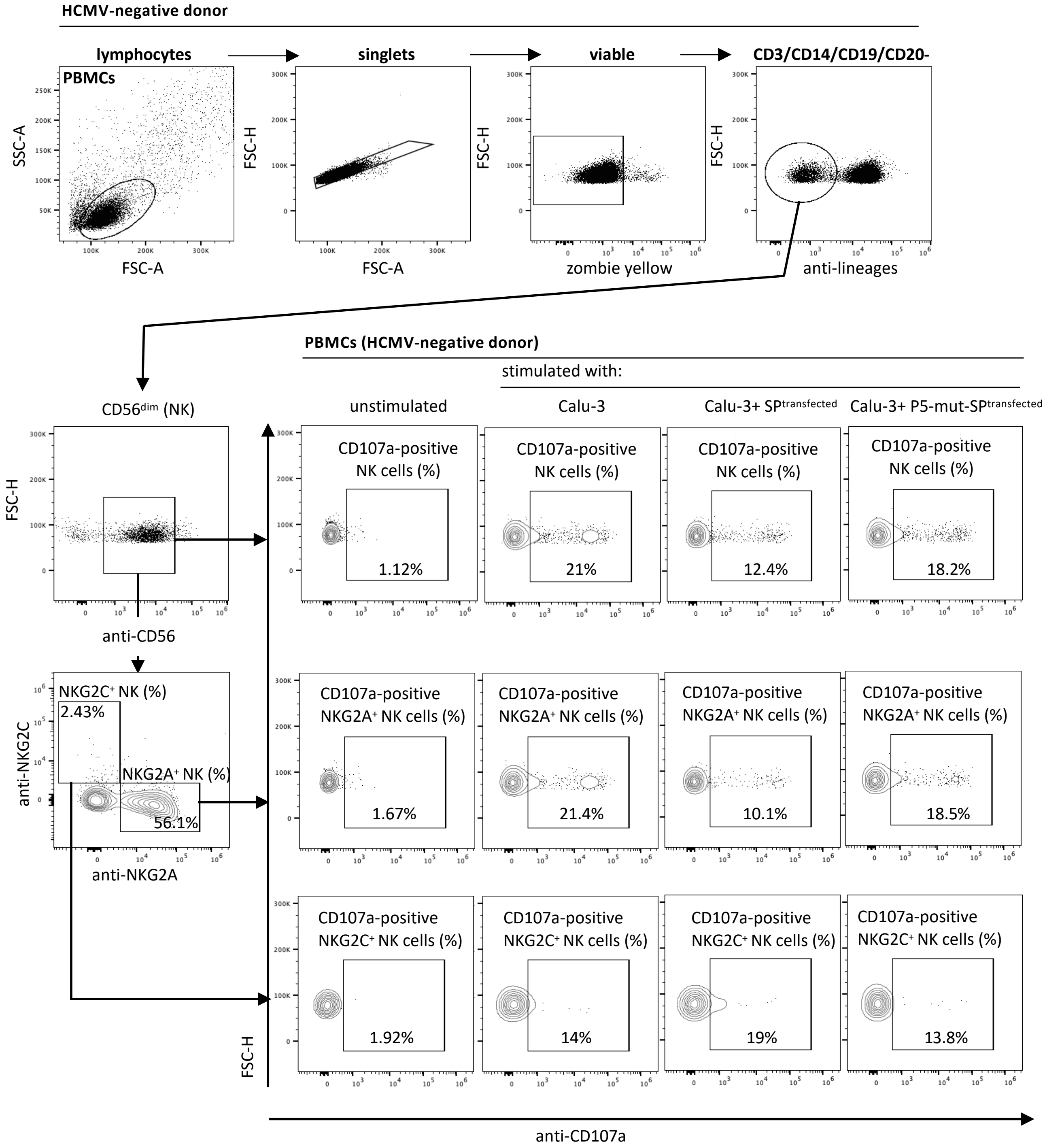

Fig. S4. Gating strategy in flow cytometry analysis of NK cells from PBMCs of a HCMV-negative donor. Top panels show gating of NK cells. Bottom panels show gating of CD56^{dim} NK cells and degranulation marker CD107a either unstimulated or stimulated with untransfected Calu-3 cells or transfected with wildtype spike protein (SP^{transfected}) or with spike protein mutated at the P5 position of peptide YLQPRTFLL in the spike protein (P5-mut-SP^{transfected}). Results of degranulation assays are presented for all NK cells, and the NKG2A+ and the NKG2C+ NK cell subpopulations.

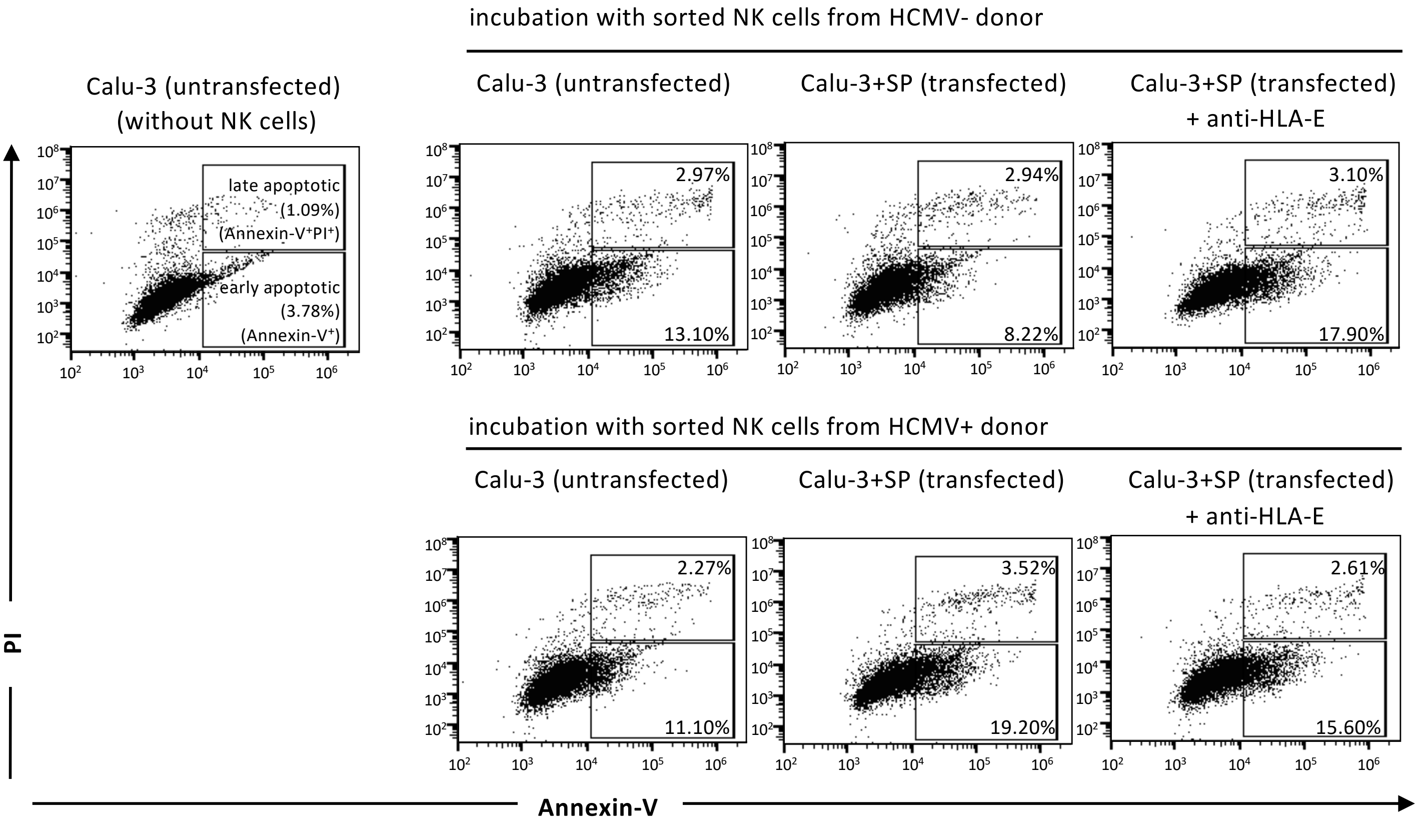

Fig. S5. Analysis of apoptosis induction in target cells expressing the SARS-CoV-2 spike protein. Flow cytometric analysis for detection of early and late apoptotic Calu-3 cells upon interaction with NK cells (lin-CD56^{dim} sorted from PBMCs of HCMV- and HCMV+ donor). Dot blots show the percentage of early (annexin V^{+}) and late apoptotic (annexin V^{+}PI^{+}) untransfected Calu-3 cells without co-incubation with NK cells as neg. con. (upper left panel). Calu-3 cells (untransfected or SP-transfected (-/+ blocking anti-HLA-E antibody 4D12)) were incubated with sorted CD56^{dim} NK cells and the percentages of early and late apoptotic cells are indicated (top and bottom panels). Co-incubations were performed at an effector:target cell ratio of 2:1.

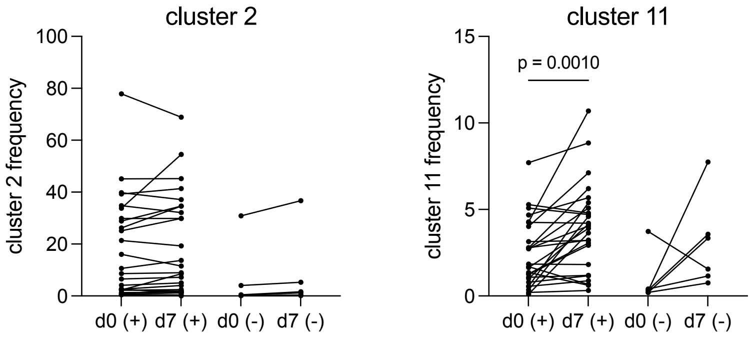

Fig. S6. Percentage of Phenograph clusters #2 and #11 in NK cells from HCMV (+) and (-) COVID-19 patients at day 0 and day 7. Sample preparation for multicolor spectral flow cytometry is described in Claus et al. (42). Samples were measured on a 5 laser Cytek® Aurora (Cytek® Biosciences), and data were analyzed using the FlowJo software (version 10.8.2; FlowJo LLC, USA) incl. the plugins FlowAI, DownSample, tSNE (optSNE), Phenograph and Cluster Explorer for high parameter analysis. Statistical analyses: Mixed-effects analysis with Šídák's multiple comparisons test.

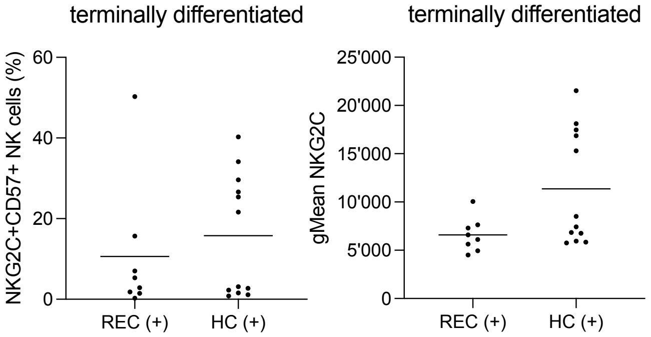

Fig. S7. Percentage of mature adaptive NKG2C+CD57+ NK cells (left panel) and cell surface expression level (geometric mean) of NKG2C on mature adaptive NK cells (right panel) from HCMV+ recovered patients (REC) and healthy controls (HC). Samples from HC were obtained before the SARS-CoV-2 pandemic.

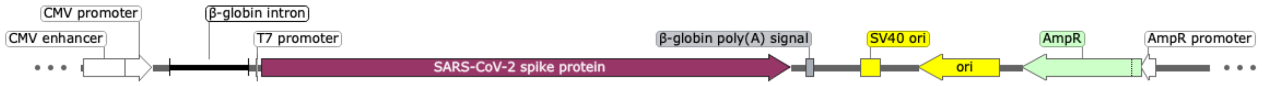

MFVFLVLLPLVSSQCVNLTTRTQLPPAYTNSFTRGVYYPDKVFRSSVLHSTQDLFLPFFSNVTWFHAIHVSGTNGTK
RFDNPVLPFNDGVYFASTEKSNIIRGWIFGTTLDSKTQSLLIVNNATNVVIKVCEFQFCNDPFLGVYYHKNNKSW
MESEFRVYSSANNCTFEYVSQPFLMDLEGKQGNFKNLREFVFKNIDGYFKIYSKHTPINLVRDLPQGFSALEPLVD
LPIGINITRFQTLLALHRSYLTPGDSSSGWTAGAAAYYVG**YLQPRTFLL**KYNENGTITDAVDCALDPLSETKCTLKSF
TVEKGIYQTSNFRVQPTESIVRFPNITNLCPFGEVFNATRFASVYAWNRKRISNCVADYSVLYNSASFSTFKCYGVSP
TKLNDLCFTNVYADSFVIRGDEVRQIAPGQTGKIADYNYKLPDDFTGCVIAWNSNNLDSKVGGNYNYLYRLFRKS
NLKPFERDISTEIYQAGSTPCNGVEGFNCYFPLQSYGFQPTNGVGYQPYRVVVLSFELLHAPATVCGPKKSTNLVK
NKCVNFNFNGLTGTGVLTESNKKFLPFQQFGRDIADTTDAVRDPQTLEILDITPCSFGGVSVITPGTNTSNQVAVLY
QDVNCTEVPVAIHADQLTPTWRVYSTGSNVFQTRAGCLIGAEHVNNSYECDIPIGAGICASYQTQTNSPRRARSV
ASQSIIAYTMSLGAENSVAYSNNSIAIPTNFTISVTTEILPVSMTKTSVDCTMYICGDSTECSNLLLQYGSFCTQLNR
ALTGIAVEQDKNTQEVFAQVKQIYKTPPIKDFGGFNFSQILPDPSKPSKRSFIEDLLFNKVTLADAGFIKQYGDCLGD
IAARDLICAQKFNGLTVLPPLLTDEMIAQYTSALLAGTITSGWTFGAGAALQIPFAMQMAYRFNGIGVTQNVLYE
NQKLIANQFNSAIGKIQDSLSSTASALGKLQDVVNQNAQALNTLVKQLSSNFGAISSVLNDILSRLDKVEAEVQIDR
LITGRLQSLQTYVTQQLIRAAEIRASANLAATKMSECVLGQSKRVDFCGKGYHLMSFPQSAPHGVVFLHVTYVPA
QEKNFTTAPAICHDGKAHFPREGVFVSNGTHWFVTQRNFYEPQIITTDNTFVSGNCDVVIGIVNNTVYDPLQPEL
DSFKEELDKYFKNHTSPDVDLGDISGINASVVNIQKEIDRLNEVAKNLNESLIDLQELGKYEQYIKWPWYIWLGFIA
GLIAIVMVTIMLCCMTSCCSCLKGCCSCGSCCKFDEDDSEPVLKGVKLHYT*

Fig. S8. Plasmid map and amino acid sequence of the spike protein used for transfection. Peptide YLQPRTFLL at positions 269-277 is shown in bold. Alanine substitutions were introduced at positions 270 and 273 to encode a spike protein with mutation at either the P2 position of the peptide (....**YAQPRTFLL**...) or the P5 position (....**YLQPATFLL**...).

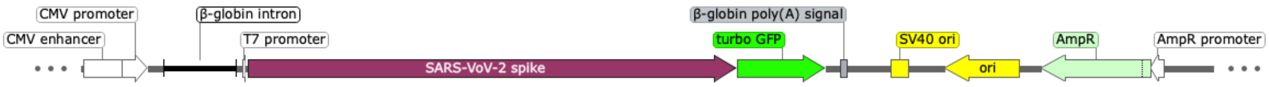

MFVFLVLLPLVSSQCVNLTTRTQLPPAYTNSFTRGVYYPDKVFRSSVLHSTQDLFLPFFSNVTWFHAIHVSGTNGTK
RFDNPVLPFNDGVYFASTEKSNIIRGWIFGTTLDSKTQSLLIVNNATNVVIKVCEFQFCNDPFLGVYYHKNNKSW
MESEFRVYSSANNCTFEYVSQPFLMDLEGKQGNFKNLREFVFKNIDGYFKIYSKHTPINLVRDLPQGFSALEPLVD
LPIGINITRFQTLLALHRSYLTPGDSSSGWTAGAAAYYVGYLQPRTFLLKYNENGTITDAVDCALDPLSETKCTLKSF
TVEKGIYQTSNFRVQPTESIVRFPNITNLCPFGEVFNATRFASVYAWNRKRISNCVADYSVLYNSASFSTFKCYGVSP
TKLNDLCFTNVYADSFVIRGDEVRQIAPGQTGKIADYNYKLPDDFTGCVIAWNSNNLDSKVGGNYNYLYRLFRKS
NLKPFERDISTEIYQAGSTPCNGVEGFNCYFPLQSYGFQPTNGVGYQPYRVVVLSFELLHAPATVCGPKKSTNLVK
NKCVNFNFNGLTGTGVLTESNKKFLPFQQFGRDIADTTDAVRDPQTLEILDITPCSFGGVSVITPGTNTSNQVAVLY
QDVNCTEVPVAIHADQLTPTWRVYSTGSNVFQTRAGCLIGAEHVNNSYECDIPIGAGICASYQTQTNSPRRARSV
ASQSIIAYTMSLGAENSVAYSNNSIAIPTNFTISVTTEILPVSMTKTSVDCTMYICGDSTECSNLLLQYGSFCTQLNR
ALTGIAVEQDKNTQEVFAQVKQIYKTPPIKDFGGFNFSQILPDPSKPSKRSFIEDLLFNKVTLADAGFIKQYGDCLGD
IAARDLICAQKFNGLTVLPPLLTDEMIAQYTSALLAGTITSGWTFGAGAALQIPFAMQMAYRFNGIGVTQNVLYE
NQKLIANQFNSAIGKIQDSLSSTASALGKLQDVVNQNAQALNTLVKQLSSNFGAISSVLNDILSRLDKVEAEVQIDR
LITGRLQSLQTYVTQQLIRAAEIRASANLAATKMSECVLGQSKRVDFCGKGYHLMSFPQSAPHGVVFLHVTYVPA
QEKNFTTAPAICHDGKAHFPREGVFVSNGTHWFVTQRNFYEPQIITTDNTFVSGNCDVVIGIVNNTVYDPLQPEL
DSFKEELDKYFKNHTSPDVDLGDISGINASVVNIQKEIDRLNEVAKNLNESLIDLQELGKYEQYIKWPWYIWLGFIA
GLIAIVMVTIMLCCMTSCCSCLKGCCSCGSCCKFDEDDSEPVLKGVKLHYTMESDESGLPAMEIECRITGTLNGVE
FELVGGGEGTPEQGRMTNKMKSTKGALTFSPYLLSHVMGYGFYHFGTYPSGYENPFLHAINNGGYTNTRIEKYE
DGGVLHVSFSYRYEAGRVIGDFKVMGTGFPEDSVIFTDKIIRSNATVEHLHPMGDNDLDGSFTRTFSLRDGGYYS
SVVDSHMHFKSAIHPSILQNGGPMFAFRRVEEDHSNTELGIVEYQHAFKTPDADAGEE*

Fig. S9. Plasmid map and amino acid sequence of the spike-turboGFP fusion protein. The turboGFP part is marked in green.

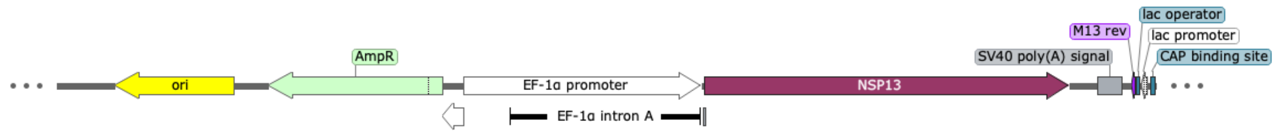

MAVGACVLCNSQTSLRCGACIRRPFLCCKCCYDHVISTSHKLVLSVNPYVCNAPGCDVTDVTQLYLGGMSYYCKS
HKPPISFPLCANGQVFGLYKNTCVGSDNVTDFNAIATCDWTNAGDYILANTCTERLKLFAAETLKATEETFKLSYGI
ATVREVLSDRELHLSWEVGKPRPPLNRNYVFTGYRVTKNSKVQIGEYTFEKGDYGDAVVYRGTTTYKLNVGDYFV
LTSHT**VMPLSAPTL**VPQEHYVRITGLYPTLNISDEFSSNVANYQKVGMQKYSTLQGPPGTGKSHFAIGLALYYPSA
RIVYTACSHAAVDALCEKALKYLPIDKCSRIIPARARVECFDKFKVNSTLEQYVFCTVNALPETTADIVVFDEISMATN
YDLSVVNARLRAKHYVYIGDPAQLPAPRTLLTKGTLEPEYFNSVCRLMKTIGPDMFLGTCRRCPAEIVDTVSALVYD
NKLKAHKDKSAQCFKMFYKGVITHDVSSAINRPQIGVVREFLTRNPAWRKAVFISPYNSQNAVASKILGLPTQTVD
SSQGSEYDYVIFTQTTETAHSCNVNRFNVAITRAKVGILCIMSDRDLYDKLQFTSLEIPRRNVATLQ*

Fig. S10 Plasmid map and amino acid sequence of the SARS-CoV-2 NSP13 protein. Peptide VMPLSAPTL is shown in bold.
